# Neurophysiological treatment effects of mesdopetam, pimavanserin and clozapine in a rodent model of Parkinson's disease psychosis

**DOI:** 10.1016/j.neurot.2024.e00334

**Published:** 2024-02-16

**Authors:** Tiberiu Loredan Stan, Abdolaziz Ronaghi, Sebastian A. Barrientos, Pär Halje, Luciano Censoni, Emilio Garro-Martínez, Azat Nasretdinov, Evgenya Malinina, Stephan Hjorth, Peder Svensson, Susanna Waters, Kristoffer Sahlholm, Per Petersson

**Affiliations:** a The Group for Integrative Neurophysiology, Department of Medical and Translational Biology, Umeå University, Umeå, Sweden; b The Group for Integrative Neurophysiology and Neurotechnology, Department of Experimental Medical Science, Lund University, Lund, Sweden; c Department of Medical and Translational Biology, Wallenberg Centre for Molecular Medicine, Umeå University, Umeå, Sweden; d Department of Physiology and Pharmacology, Karolinska Institutet, Solna, Sweden; e Integrative Research Laboratories Sweden AB, Göteborg, Sweden

**Keywords:** Antipsychotics, In vivo, Behavior, Local field-potentials, High-frequency oscillations

## Abstract

Psychosis in Parkinson's disease is a common phenomenon associated with poor outcomes. To clarify the pathophysiology of this condition and the mechanisms of antipsychotic treatments, we have here characterized the neurophysiological brain states induced by clozapine, pimavanserin, and the novel prospective antipsychotic mesdopetam in a rodent model of Parkinson's disease psychosis, based on chronic dopaminergic denervation by 6-OHDA lesions, levodopa priming, and the acute administration of an NMDA antagonist. Parallel recordings of local field potentials from eleven cortical and sub-cortical regions revealed shared neurophysiological treatment effects for the three compounds, despite their different pharmacological profiles, involving reversal of features associated with the psychotomimetic state, such as a reduction of aberrant high-frequency oscillations in prefrontal structures together with a decrease of abnormal synchronization between different brain regions. Other drug-induced neurophysiological features were more specific to each treatment, affecting network oscillation frequencies and entropy, pointing to discrete differences in mechanisms of action. These findings indicate that neurophysiological characterization of brain states is particularly informative when evaluating therapeutic mechanisms in conditions involving symptoms that are difficult to assess in rodents such as psychosis, and that mesdopetam should be further explored as a potential novel antipsychotic treatment option for Parkinson psychosis.

## Introduction

Parkinson's disease (PD) associated psychosis (PD-P) is a common phenomenon associated with poor outcomes [1]. It is associated with increased caregiver distress and is a leading reason for nursing home placement of people with PD [2]. While the epidemiology of PD-P remains uncertain due to differences in the reporting of symptoms, the methods of assessment and the selection of patients, data nevertheless indicate that visual hallucinations are estimated to be present in more than one quarter of people with PD [3,4]. Other phenomena, such as auditory hallucinations, presence hallucinations, and delusions are less prevalent but taken together PD-P-related complications often severely reduce the quality of life for patients [3]. Endogenous risk factors for PD-P include cognitive impairment, older age/longer duration of PD, and disease severity. Notably, dopamine replacement therapy often aggravates symptoms [3,5]. While most available antipsychotics can reduce PD-P symptoms, tolerability concerns, especially the worsening of the motor symptoms of PD, render almost all of them difficult to use. The atypical antipsychotic clozapine has been shown to improve symptoms in PD-P without significantly worsening the motor symptoms and is consequently regarded as a clinically useful treatment option [4,6]. However, in addition to clozapine's risk of generating agranulocytosis, atypical antipsychotics must be used with great caution in demented patients with psychosis because of risk of adverse effects such as falls, worsening of cognitive function, cardiovascular effects, stroke, and death [4,7]. In this context, pimavanserin, a selective 5-HT2A antagonist/inverse agonist without activity at dopamine receptors has certain advantages compared to other treatments and has received regulatory approval for symptomatic treatment of visual hallucinations and delusions in PD-P [8]. However, recent analyses of real-world data from the clinical use of pimavanserin suggest that while pimavanserin is safe and tolerable, relatively few patients experience robust improvement of their symptoms in the long term [9]. Thus, improved treatments for PD-P remains an urgent clinical need. In the search for novel treatments for PD-P, certain dopamine receptor sub-types remain possible targets. For example, in rodents, non-human primates, and humans, long-term treatment with levodopa has been associated with an increase in dopamine D3 receptor (D3R) expression in parts of the brain known to be specifically affected in PD, such as striatum and globus pallidus [[10], [11], [12], [13]]. Moreover, D3Rs have also been implicated as a relevant target for antipsychotic treatment in schizophrenia [14]. Thus, it is possible that pharmacological suppression of D3R-signaling could help ameliorate side effects of chronic levodopa treatment, including symptoms of PD-P. Mesdopetam is a dopamine type-2-receptor antagonist with a strong preference for D3Rs and more agonist-like physicochemical properties than other D3R antagonists. An agonist like binding mode at D3Rs has been proposed to contribute to its *in vivo* pharmacological profile as well as the good tolerability in patients with PD [[14], [15], [16], [17]]. In recent years, methods for recording activity in widely distributed brain circuits have developed rapidly and have been adapted to the small brains of rodents [18,19]. These technologies offer a unique opportunity to peer into the processes that underlie cognitive and emotional states, and importantly, provide a powerful tool with which to analyze the effects of pharmacological substances. Hence, to clarify if mesdopetam has the potential to be used as treatment for PD-P, we have here directly compared the treatment effects of mesdopetam, pimavanserin, and clozapine in a rat model of PD-P, with respect to both behavioral and neurophysiological effects induced by the three drugs, using chronic recordings of brain activity in freely behaving animals. Since mesdopetam is thought to primarily act via D3Rs, we have also performed experiments with a selective D3R-antagonist, SB277011-A [20], as a reference. Importantly, brain activity was recorded from a large group of brain structures in parallel, making it possible to characterize the pharmacological effects on brain activity on a system level [19].

## Methods

### Animals and housing

Adult female Sprague-Dawley rats were used (250–350g, approximately four months old; Taconic Biosciences, Denmark for Lund experiments and in-house colony in Umeå). Animals were housed at Umeå Center for Comparative Biology's (UCCB, n ​= ​3) and at Medicon Village, Lund University (n ​= ​5). Animals were maintained at a temperature of 22–24 ​°C in a 12 ​h light/dark cycle, with food and water available *ad libitum*. National and local guidelines for animal welfare were followed and all procedures were approved in advance by the Swedish Ethical Committee for Northern Sweden (Dnr. A15-2018) and by the Malmö/Lund ethical committee (Ethic permit number 9/18 01689/2019) of animal experiments, respectively.

### 6-OHDA lesions and procedures for surgical implantations

For 6-OHDA lesions, animals were anesthetized with Fentanyl/Medetomidine (Domitor®) 0.2 ​mg/kg and 0.1 ​mg/kg, respectively, given intraperitoneally (i.p) or subcutaneously (s.c.). Non-steroidal anti-inflammatory Carprofen (5 ​mg/kg, s.c.) was administered to relieve peri- and postoperative pain and attenuate the inflammatory response before surgery and up to postoperative day 5. The animals received two stereotaxic injections of 6-hydroxydopamine (6-OHDA) hydrochloride (Sigma-Aldrich, Sweden), 6.0 ​μg/μl free base dissolved in 0.02% ascorbate saline, into the medial forebrain bundle of the right hemisphere. The first injection, 2.5 ​μl, was introduced (1 ​μl/min) at the following coordinates from bregma and cortical surface: AP: 4.0, ML: 1.2, DV: 7.8, with tooth bar set at −4.5. The second injection, 2 ​μl, was applied (1 ​μl/min) at the coordinates from bregma: AP: 4.0, ML: 0.8, DV: 8.0 with tooth bar set at +3.4, following established protocols [21].

For electrode implantations, anesthetic/analgesic procedures and postoperative care were similar to 6-OHDA lesions. In addition, to minimize the risk of infection, the antibiotic Enrofloxacin (5 ​mg/kg, s.c.) was administered 2 ​h before the surgery and up to postoperative day 7. During surgery, microwire electrodes were implanted in both hemispheres, using stereotaxic procedures. The implant was fixated with dental acrylic, which was attached to anchoring screws (Agntho's AB, Lidingö, Sweden, Ø 2 ​mm ​× ​3 ​mm each) inserted in the skull. A 200 ​μm thick silver wire was attached to the posterior skull screws and used as a ground connection from the animal to the recording system. For the first three postoperative hours, animals were placed on a heating blanket. Postoperative analgesia was provided following the postoperative day 1–5 according to local guidelines. The animals were allowed to recover for at least two weeks after surgery before initiation of experimental protocol.

### Recording electrodes

Recording arrays were built according to the procedure previously described by Ivica et al. [18]. Formvar-insulated tungsten wires (33 ​μm diameter, California Fine Wire Co., CA) were arranged into 30 groups of arrays (15 groups per hemisphere targeting different brain areas with 2–8 wires per target) with 250 ​μm wire spacing in each horizontal dimension and a wire length corresponding to the depth of the recording target. After verification of tip locations with CT [21], four structures (claustrum, insula, dorsal hippocampus and mediodorsal thalamus) were eliminated because they were not sufficiently covered across animals (criterion used: recordings from both left and right hemisphere from at least 3 animals; see also Supplementary Fig. 1). The remaining 11 regions that were used throughout the study were (with abbreviations used in parenthesis): amygdala (amyg), dorsal striatum (dStr), olfactory cortex (OC), orbitofrontal cortex (OFC), parietal cortex (PC), medial prefrontal cortex (mPFC), primary motor cortex (M1), primary somatosensory cortex (S1), thalamus (thal), ventral hippocampus (vHipp), ventral striatum (vStr). A full list of coordinates within each of these structures (and the validated electrode positions, n ​= ​634) is appended as Supplementary Data.

### l-DOPA priming procedure

**Fig. 1:**
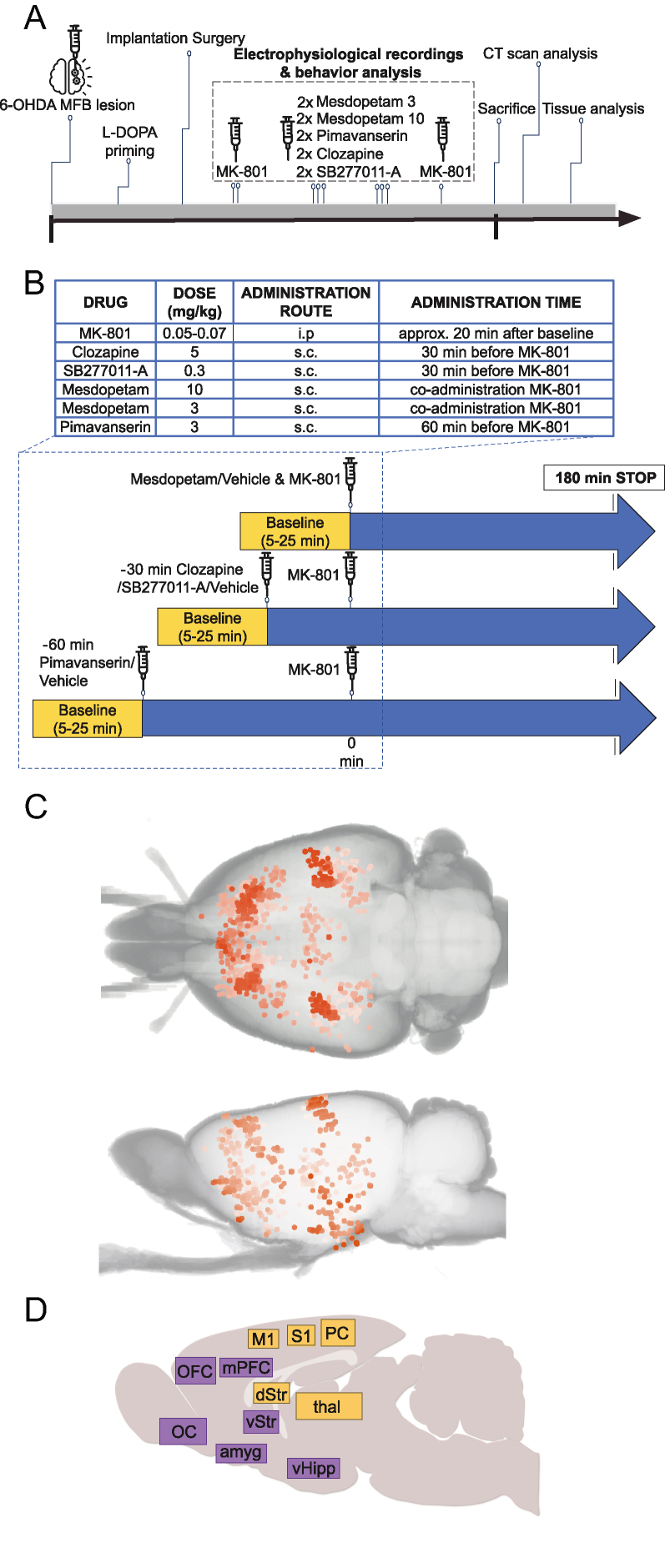
**Overview of experiment design and brain structures recorded**. A) Experimental timeline (period from lesion to sacrifice corresponds to approximately 3 months). B) Summary of doses, and administration routes/times for the compounds investigated. C) Overview of tip positions for all recording electrodes, as reconstructed from postmortem CT scans (overlapping sites are indicated by higher color saturation [21]. D) Summary of anatomical locations analyzed; colors mark structures with predominantly cognitive/limbic (purple) versus sensorimotor (yellow) related functions.

When fully recovered after lesion procedure (14–16 days after injection), animals were treated with daily doses of levodopa (l-DOPA)/benserazide (Sigma-Aldrich, Sweden), dissolved in saline and administered 10/7.5 ​mg/kg i.p. [22], for 14 days in total (Fig. 1A). Signs of hyper-/dyskinesia were monitored and assessed by a highly trained experimenter determining duration and severity of axial/limb/orofacial abnormal involuntary movements for 1 ​min every 10 ​min. All animals that showed moderate to high levels of dyskinetic symptoms (i.e. displayed axial, limb and orolingual abnormal involuntary movements corresponding to a score of two or higher on the scale defined by Cenci & Lundblad [23]) were implanted with an electrode array.

### Pharmacological experiments

At least two weeks after the electrode array implantation, animals were subjected to pharmacological treatments during electrophysiological and behavioral recordings (Fig. 1A and B). In brief, vehicle experiments were performed first and last in each experimental series and experiments involving pharmacological treatments were performed in a randomized order for each rat. Experiments were performed at least two days apart in each rat over a total period of up to five months. Drugs were administered in a bolus volume of 1.0 ​ml/kg.

The selective and non-competitive NMDA receptor antagonist, (+)MK-801 maleate (Tocris, Sweden; Sigma-Aldrich) was diluted in saline and administered i.p., 20 ​min after the baseline at a dose (0.05–0.07 ​mg/kg) previously shown to induce psychosis-like episodes as judged by changes in EEG oscillatory activity [24]. The atypical antipsychotic clozapine (Tocris, Sweden) was administered s.c. 30 min prior MK-801 administration at a dose of 5 ​mg/kg, as previously reported [25]. The selective D3R antagonist SB277011-A dihydrochloride (Tocris, Sweden) was administered s.c. 30 min prior to MK-801 administration at a dose of 0.3 ​mg/kg, as previously reported [26]. For mesdopetam (provided by Integrative Research Laboratories Sweden AB, Gothenburg, Sweden) we used two different doses, 3 ​mg/kg and 10 ​mg/kg [17]. Mesdopetam was diluted in saline and delivered s.c. and administered jointly with MK-801. The selective 5-HT2A antagonist/inverse agonist, pimavanserin (AbMole Bioscience, Sweden) was administered s.c. (3 ​mg/kg) 60 ​min prior to MK-801 administration. The pimavanserin dose used has previously been shown to reverse psychosis-like behaviors in a rodent model of PD [27].

### Behavioral and electrophysiological recordings during experimental anti-psychotic treatment

Behavioral and electrophysiological recordings were performed in a Faraday cage. Animals were placed in a transparent acrylic cylinder (50–60 ​cm in diameter). Digital cameras filmed the rat from the side, as well as either from above or below. Video frames were acquired at 25 fps triggered by a pulse generator (Master-8/9, AMPI, Israel), which enabled synchronization to electrophysiological recordings. For an overview of the experimental protocol, see the schematic picture (Fig. 1B). In brief, after acclimatization for 10 ​min to the recording environment, animals were initially recorded during baseline conditions for 20 ​min (baseline data were sampled 5–25 ​min from the start of the recording) followed by recording under different pharmacological treatment conditions. The time between MK-801 administration and antipsychotic treatment was adapted to the pharmacodynamic profile of each drug. Recording of electrical activity and locomotor activity continued up to 180 ​min after MK-801 injection (Fig. 1B; treatment data were collected within 40–60 ​min after injection of MK-801).

### Automated quantification of spontaneous motor behavior

Video recordings were processed to extract coordinates for selected body parts using DeepLabCut [28,29]. In brief, separate networks were trained for the top and bottom camera using default settings and a minimum of 600 manually labeled frames each. The network was then applied to extract coordinates from all video recordings. Tracked body parts included tail base, tail tip and body center, as well as left and right front and hind paws and mouth for the bottom camera network or left and right ear, top of head and nose for the top camera network.

The position of the animal was determined by averaging the xy coordinates of all body parts. Subsequently, we computed several key analysis metrics during the observation periods (5–25 ​min during baseline, and 40–60 ​min after MK-801 administration). Speed was determined by calculating the positional difference between consecutive time frames, t(n) and t(n+1). To evaluate specific movement characteristics during the recordings, we identified locomotion bouts characterized by a minimum speed of 2 ​cm/s and a duration of at least 0.5 ​s from the speed profile of each animal. We then compared various parameters, including average speed, distance, duration, and frequency of detected bouts within the observation periods.

### Neurophysiological signal acquisition and processing

Electrical signals were filtered to obtain signals between 1 and 7500 ​Hz, sampled and digitized at 30 ​kHz by the amplifier in the headstage, and recorded by the Intan RHS2000 and RHD2000 systems. Signals were subsequently down-sampled to 2000Hz, off-line.

Bipolar local field potential (LFP) time series were computed from separate pairs of electrodes located in the same structure. By using this method, we made sure that external sources contributed minimally to the local voltage fluctuations via volume conductance, thus better separating out the local sources. Time-frequency power spectral densities (i.e. spectrograms) were calculated over the 0–300 ​Hz frequency range with 50%-overlapping 8-s Hanning windows (0.5 ​Hz resolution) for each of the time series. To emphasize oscillatory components in the power spectrum, we used irregular resampling [30]. The power spectrum of arrhythmic components is unaffected by time series resampling, thus enabling the separation of the fractal component of the spectrum by resampling the time series multiple times. Then, by normalizing the total spectrum to the fractal component, it is possible to construct a power spectrum measure that emphasizes truly rhythmic activity.

### Peak detection

To detect high-frequency oscillations (HFOs) and gamma bands oscillations, we defined a parametric model,

y(f)=A∗exp(−((f−B)/C)2)+Df+E

which was fitted (Matlab fit function) to the spectra obtained by averaging together the spectra of individual electrode pairs from the same structure. The parameters A (peak height), B (peak frequency), C (peak width), D (inclination of flat background), and E (offset of flat background) were estimated such that the model y(f) fitted the spectrum optimally in the least-squares sense. This allowed us to detect HFO/Gamma peaks automatically by defining thresholds for the goodness-of-fit (R2) and the fitted parameters. Typical conditions for a positive detection were R2>0.2, 2 ​< ​A<100 ​dB, 115 ​< ​B ​< ​170 ​Hz (HFO) or 30 ​< ​B ​< ​70 (gamma), 1 ​< ​C ​< ​20 ​Hz, −1<D ​< ​1 and −10 ​< ​E ​< ​10. In the event of positive detections, the model was also used to quantify peak frequency parametrically.

Band power was calculated as the mean power over a 20 ​Hz band centered on the peak frequency. The peak frequency was defined individually for each recording session and each structure as the median of the B parameter across the recording session.

### LFP signal complexity

LFP signal complexity was estimated through calculation of Permutation Entropy, following the procedure described in Refs. [31,32]. Signals were collected from individual recording electrodes in 10-min windows during baseline and at +40 ​min after MK-801 administration. Signals were transformed into Permutation Entropy time series using order-6 patterns of consecutive values (no delay), with a sliding window of 30,000 points (equivalent to 15 ​s), and then averaged into one representative entropy value per channel and condition. Violin plots were created using the method by Bechtold et al. [33].

### Brain state comparisons based on spectral correlations

For spectral correlation analysis, signals were collected from individual recording electrodes in 10-min windows during baseline and at +40 ​min after MK-801 administration. Spectra in the range 1–300 ​Hz from multiple structures were then normalized to the aperiodic part and concatenated. For each treatment, Pearson pairwise correlations were first calculated in relation to baseline and vehicle. Second, correlations of treatments were calculated with respect to mesdopetam (i.e. mesdopetam vs. clozapine, pimavanserin and SB-277011-A).

### X-ray tomography to verify electrode positions

To verify the accurate anatomical targeting of the studied brain structures, we utilized a newly developed semi-automated method, employing acquired Computer Tomography (CT) images [21]. After the completion of recording experiments, the rats were anesthetized with a lethal dose of sodium pentobarbital (100 ​mg/kg i.p., Apoteksbolaget AB, Sweden). Following transcardial perfusion with 0.9% saline and subsequent fixation with 4% paraformaldehyde, the animals were decapitated *postmortem*. The heads, along with the preserved electrode implants, were then immersed in 4% paraformaldehyde for 24 ​h before being transferred to a 25% sucrose solution to prevent excessive tissue dryness and shrinkage. CT scans were conducted using the MILabs XUHR system (MILabs, the Netherlands) and Mediso Nanoscan PETCT scanner (Mediso, Hungary), with the heads positioned perpendicular to the photon beam [21]. The scanned volumes were registered to an anatomical atlas using bone landmarks and the image coordinates of the electrode tips were converted to stereotaxic atlas coordinates. Finally, the wire tips were assigned appropriate anatomical labels based on their location in the atlas [34].

### Statistical analyses

For locomotion analyses, statistical significance between the specific time periods of the task was tested for each drug using a generalized linear mixed effect model (GLMM). The GLMM formula included main effects for ‘Drug’ and ‘Period,’ their interaction, and random effects for ‘Rat’ nested within ‘Session’. An analysis of variance (ANOVA) was performed on the GLMM model to assess the significance of the main effects and interactions. Subsequently, upon significant omnibus ANOVA, pairwise comparisons for either between treatments during peak period or between periods for each treatment were conducted. Dunnett's adjustment for multiple comparisons was applied, and confidence intervals at a 95% level were computed. Oscillation detection rate, band power and frequency were compared to baseline with a nested ANOVA model independently for each structure with factors State (baseline vs drug), Session and Animal. The factor Session was nested in Animal. Session and Animal were defined as random variables. The model was identical for comparisons between drugs (for example, MK-801+vehicle vs. MK-801+clozapine), except that Session was nested in both Animal and State.

For LFP signal complexity values, for each structure, distributions of values from each condition were independently compared against baseline and against MK-801+vehicle using nested N-way ANOVA, with Animal, Session, Channel and Condition as factors, such that Session and Channel were nested under Animal. For comparisons against MK-801+vehicle, Session was also nested under Condition. P-values <0.05 were reported as significant. For the global state descriptors based on spectral content across structures, non-parametric tests were used based on average data per animal. All analyses were performed in Matlab.

## Results

### A rodent model of PD-P allowing for systems-level neurophysiological investigations

To compare the behavioral and neurophysiological treatment effects of mesdopetam to the two existing antipsychotics regarded as clinically useful for the treatment of PD-P, eight rats that had previously been exposed to unilateral 6-OHDA lesions to the medial forebrain bundle and had been primed with levodopa according to previously validated protocols used to model clinical PD with dyskinesia [[35], [36], [37]], were injected with the NMDA receptor antagonist MK-801 (27); Fig. 1A [24,27]. In conjunction with MK-801 administration, animals were treated with mesdopetam, pimavanserin, or clozapine (for simplicity, all three substances are here collectively referred to as antipsychotic compounds), each compound in at least two separate experimental sessions, during which behavior and neurophysiological changes in brain activity patterns were recorded and compared to control experiments using MK-801+vehicle injections (Fig. 1A and B; in total 391 ​h of electrophysiological recordings were analyzed).

As cognitive processes are thought to involve widely distributed brain networks [38], it was of particular importance to obtain brain activity recordings on a large scale, encompassing several individual brain structures. To this aim, we developed specialized chronic implants that enable individual targeting of 128 separate recording sites arranged in complex geometries [18]. By analyzing 3D reconstructions from CT-scanned heads [21] we could verify that a total of 914 recording electrodes were distributed in 115 different brain structures [34], divided approximately equally between the two hemispheres (Fig. 1C). Based on functional relatedness and sufficient coverage across animals recording sites were merged into 11 groups for further analyses (Fig. 1D; Supplementary Figure 1; Supplementary Data).

### Characterization of behavioral features revealed behaviors associated with psychosis models but modest treatment effects of antipsychotic compounds

**Fig. 2:**
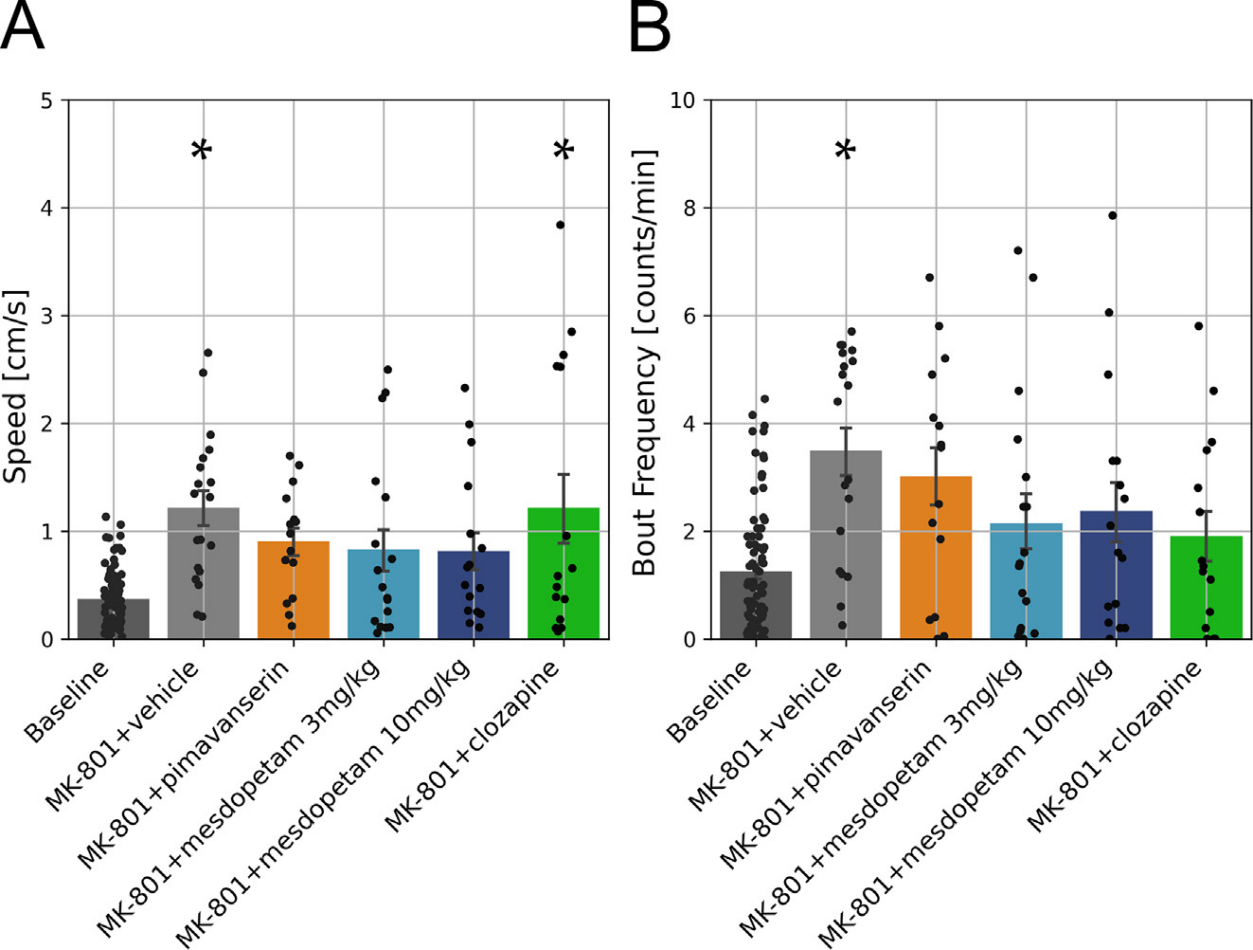
**Quantification of spontaneous behavior in the open-field**. A) Average locomotion displayed per second. B) Frequency of locomotion bout initiation. Black dots correspond to individual recording sessions, and asterisks denote significant differences with respect to baseline (p ​< ​0.05, ANOVA). Error bars indicate standard error of the mean.

In parallel with the neurophysiological recordings, the spontaneous motor behavior of the rats was characterized. In lesioned rats, low-dose MK-801 proved sufficient to induce behavioral features that have previously been described as characteristic of rodent models of psychosis. That is, in agreement with previous studies, we observed a significant increase in spontaneous locomotor behavior (Fig. 2A; p ​< ​0.001; [ [39]). Specifically, compared to baseline condition, rats displayed bouts of spontaneous locomotion more frequently during the peak dose period (Fig. 2B; p ​< ​0.01; 40–60 ​min after MK-801 administration), whereas the bout length and locomotion speed per bout were relatively unchanged (p ​> ​0.9 and p ​> ​0.6, respectively; data not shown). In hemi-parkinsonian animals, a slight side-bias in spontaneous locomotor behavior may be expected [40]. A significant side-bias in spontaneous turning (ipsiversive to the side of the lesion) was indeed detected on baseline (p ​< ​0.001, *t*-test of cumulative angle difference compared to zero) and this effect was significantly increased in the more active motor state induced by MK-801 (p ​< ​0.01; ANOVA test of cumulative angle difference), however this increase in spontaneous turning was not consistently reversed by the antipsychotic treatments. Thus, although all three antipsychotic compounds tested showed tendencies to normalize excessive locomotion, none of them resulted in significant effects when compared to MK-801+vehicle during the peak-dose period (Fig. 2A and B). Accordingly, the behavioral assessments indicated drug-induced changes largely in line with previous reports but anti-psychotic treatment effects showed relatively large variability and did not reach statistical significance in this study.

### MK-801 induces high-frequency oscillatory activity in widely distributed brain structures

**Fig. 3:**
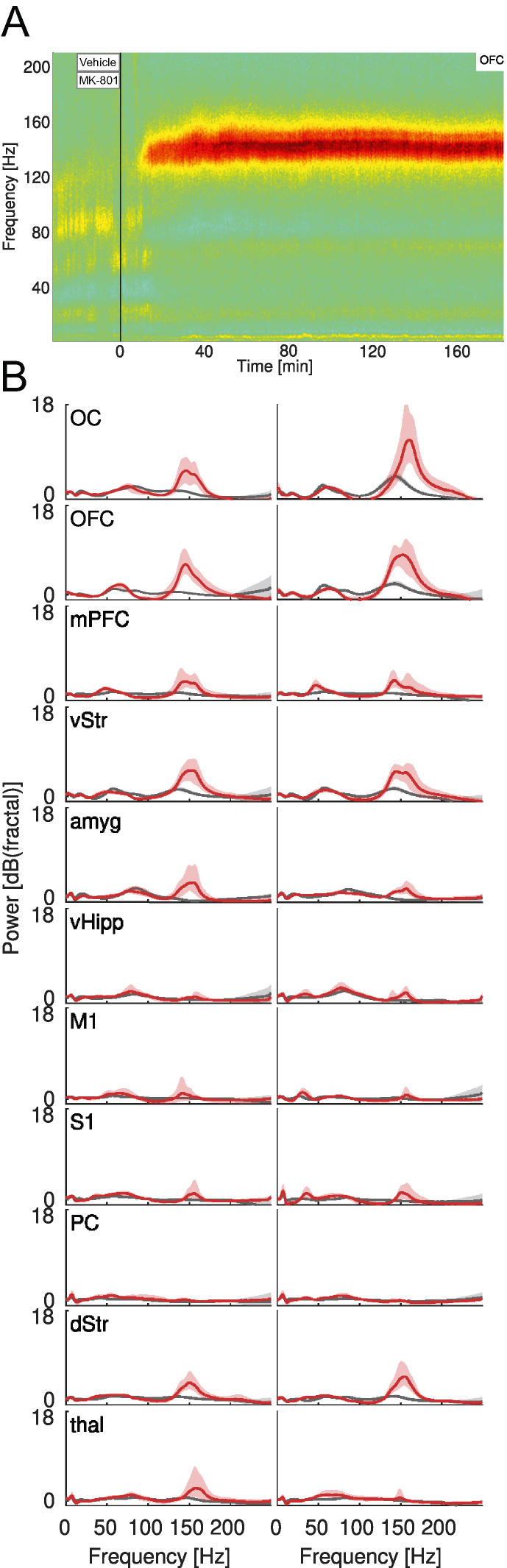
**Example of LFP data collection during an experiment and spectral features of the MK-801 PD-P model**. A) Example mean spectrogram from a single recording constructed by averaging spectrograms obtained from all differential LFP signals recorded from electrodes in OFC in the lesioned hemisphere. The spectrogram is normalized to the arrhythmic background (colors denote relative power [dB_fractal_]). The vertical line marks the administration time for MK-801+vehicle. B) Spectra obtained by averaging all differential LFP signals from electrode pairs in the corresponding structures, across all animals and recordings. The spectra are normalized to the arrhythmic background. Gray: baseline. Red: MK-801+vehicle. Left column: Intact hemisphere. Right: lesioned hemisphere.

To acquire a more complete view of the compounds' pharmacological mechanisms of action, we next sought to clarify how the different treatments affect neurophysiological brain states. In each experiment, a baseline recording was followed by MK-801 administration in combination with either vehicle or one of the antipsychotic compounds. To emphasize periodic components in the recorded signals, the spectral content was first normalized to the arrhythmic background [41]. In Fig. 3A, the resulting spectrogram from an example recording is shown, depicting the MK-801-induced changes in spectral power of the average of the differential LFP signals from all electrode pairs located in OFC in one hemisphere. Before investigating the various treatment effects in further detail, the MK-801-induced brain state was first comprehensively characterized. When pooling data across all animals and recordings, it became apparent that oscillatory activity was generally present in two frequency intervals, ranging roughly between 30-90 ​Hz and 130–160 ​Hz, respectively (Fig. 3B). We here use the terms ‘gamma oscillations’ and ‘high-frequency oscillations’ (HFOs) to refer to these two types of oscillations. Interestingly, oscillations in these two parts of the frequency spectrum have previously been implicated in relation to motor and non-motor signs, respectively, in animal models of levodopa-induced dyskinesia [[42], [43], [44], [45]]. Moreover, exaggerated activity in the HFO band is a frequent observation following administration of various psychotomimetic drugs [[46], [47], [48], [49], [50], [51]]. The widespread synchrony created by the HFOs across cognitive-limbic structures has been proposed to interfere with integration of information causing hallucinations and delusions [52]. In quantitative terms, we found that MK-801, on average, increased both HFO power (+3.2 ​dB, p ​< ​0.001, nested ANOVA) and gamma power (+0.3 ​dB, p ​< ​0.001, nested ANOVA) compared to baseline. However, since the HFO power increase was an order of magnitude larger than gamma we primarily focused our further analyses on the HFOs (for completeness, analyses of gamma band oscillations are presented in Supplementary Figure 2). Interestingly, when comparing the effects of MK-801, for the intact and lesioned hemisphere separately, it was noted that the lesioned hemisphere generally had somewhat higher HFO power, both during baseline recordings (lesioned/intact: +0.13 ​dB, p ​< ​0.001, nested ANOVA) and during MK-801 treatment (+0.16 ​dB, p ​< ​0.001, nested ANOVA), possibly suggesting an increased propensity for the generation of endogenous psychosis-associated brain activity under conditions of severe dopamine depletion following levodopa priming (details on the temporal pattern of the emergence of HFOs following MK-801 are included in Supplementary Figure 3). Overall, however, the increase in HFO power was clearly present in both hemispheres and largely involved the same structures (Fig. 3B).

### Antipsychotic treatments modify consistency, power and frequency of HFOs

**Fig. 4:**
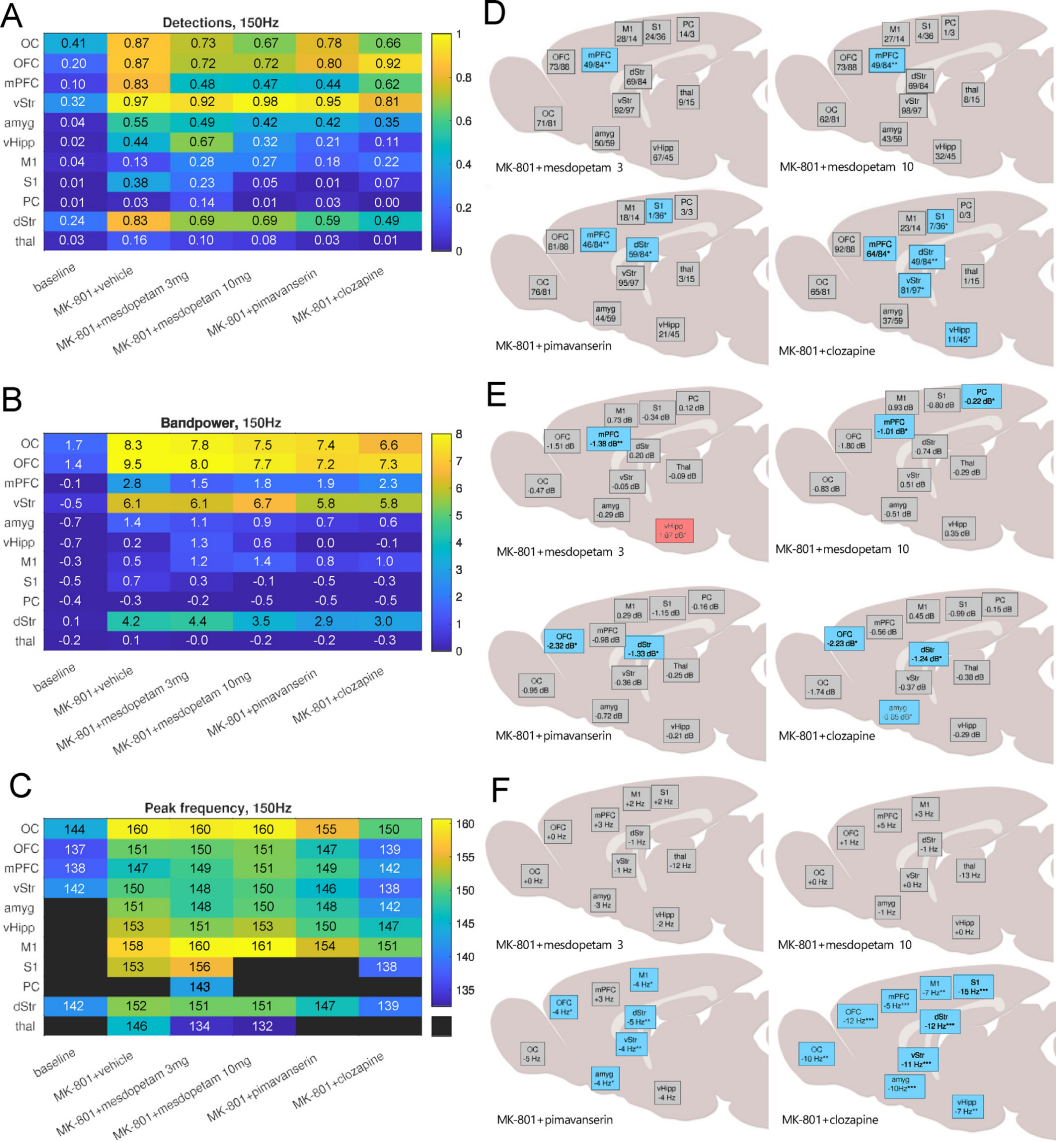
**Summary of treatment effects on high-frequency oscillation features**. A) Detection rate B) Band power (dBfractal) C) Peak frequency (Hz). D) Map of significant changes in HFO detection rate in the lesioned hemisphere (%). E) Map of significant changes in HFO band power in the lesioned hemisphere (dB_fractal_). F) Map of significant changes in HFO peak frequency in the lesioned hemisphere (Hz). Black fields in panel C denote structures where a distinct peak was absent. Blue/red squares in panels D–F mark significant reductions/increases compared to MK-801+vehicle.

Interestingly, all three antipsychotic compounds proved to modify the MK-801-induced HFOs, although not in an identical manner. Three features of the HFOs were analyzed in detail: 1) detection rate, 2) band power, and 3) peak oscillation frequency. All three compounds tended to alter these features in a graded manner in the different recorded structures. We first noted that both HFO detection rate and band power were partially reversed toward baseline conditions compared to MK801+vehicle by the different antipsychotic treatments (Fig. 4A and B). Because differences between hemispheres in comparison to the global treatment effects were comparatively small, only data for the lesioned hemisphere are presented (the corresponding data for the intact hemisphere are included in Supplementary Fig. 4). While a general tendency for HFO suppression was noted, treatment effects nevertheless differed somewhat across brain structures for the different drugs. Hence, to facilitate comparisons of heterogeneous treatment effects on a broader scale, significant changes compared to MK801+vehicle in HFO detection rate and band power were spatially mapped for the 11 recording sites, indicating that HFO suppression was most consistent in prefrontal structures (Fig. 4D and E). The corresponding analysis for peak frequencies of the HFOs revealed that clozapine markedly decreased HFO peak frequency in practically all recorded structures, whereas this effect was not as evident for the other treatments, although significant decreases were detected also for pimavanserin in some structures (Fig. 4C,F [see also ref [53]]). Taken together, one specific treatment effect was identified across all three types of antipsychotic treatments – a significant reduction in HFO detection rate in mPFC. At the same time, each drug also displayed several selective effects with respect to pattern of changes in HFO detection rate, band power and frequency, for example when comparing cortical vs. subcortical or cognitive-limbic vs. sensorimotor structures. Thus, to the extent that HFOs can be used as a neurophysiological biomarker of psychosis, our data suggest that the HFO reductions in mPFC common to all three drugs may be particularly important to achieve an antipsychotic effect, whereas differences in binding profiles to different dopaminergic and serotonergic receptors most likely can explain the drug-specific changes in brain activity patterns.

### Antipsychotic treatments modify functional connectivity and the temporal entropy of brain activity

**Fig. 5:**
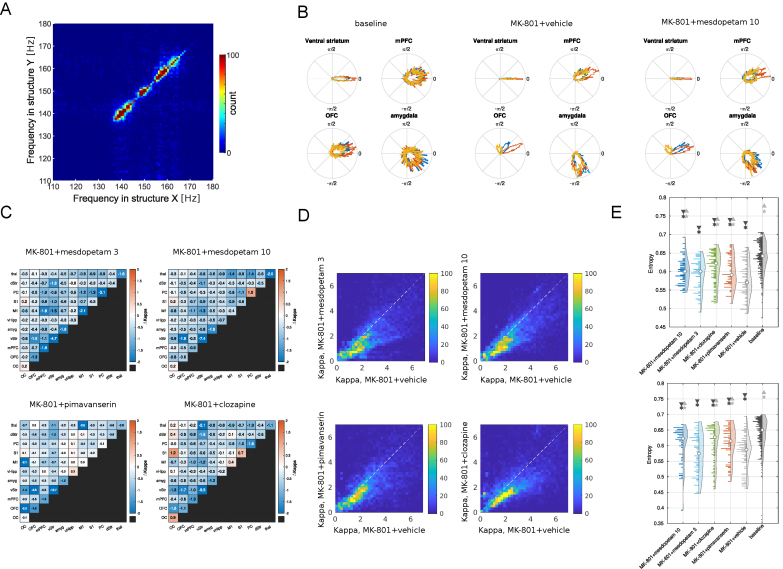
**Changes in functional connectivity and brain state entropy**. A) Histogram of HFO peak frequency for all episodes where there is simultaneous detection of HFOs in more than one structure. The marked absence of off-diagonal points demonstrates that when simultaneous HFOs are detected, they share the same frequency. B) Distributions of phase relations between 12 example electrodes (three in each structure, color coded in red, blue and orange; located within vStr, and in mPFC, OFC and amyg, respectively) and a reference electrode located in vStr, under three different treatment conditions. C) Changes in phase-synchronization (kappa-values) for electrodes located in different pairs of structures (as indicated by the x- and y-labels) for four treatment conditions compared to MK-801+vehicle (cold colors denote reduced phase-coupling). D) Scatterplot of phase synchronization (kappa values; colorbar denotes counts) for all pairs of electrodes during MK-801+vehicle (x-axis) vs. each of the four treatment conditions. Note a similar reduction in phase-coupling across the different treatments affecting electrode pairs with relatively stronger and weaker synchronization to a comparable degree. E) Distributions of permutation entropy values of the LFP signal for all electrodes in OFC and vStr in the lesioned hemisphere, pooled across animals and recordings, for different conditions. Top: OFC. Bottom: vStr. Dark gray ∗ (asterisk) denotes a significant difference from baseline, and light gray ⋆ (star) denotes a significant difference from MK-801+vehicle. Triangles point to the direction of the significant difference between means.

An intriguing feature of the analyzed HFOs is that they generally occur in several brain structures in parallel and share a common oscillation frequency at any given moment in time, indicating a high degree of functional coupling between widely distributed networks (Fig. 5A). Indeed, it has been hypothesized that this type of increased functional coupling may be a distinguishing pathophysiological feature of PD, regarding both motor and non-motor aspects of the disease [45,54]. Thus, we next investigated to what extent the exaggerated functional coupling was affected by the various treatments, using a metric based on the observed phase relations of HFOs detected, in parallel, in different recording electrodes. This is exemplified in Fig. 5B, where the distributions of phase relations between 12 example electrodes (located three each in vStr, mPFC, OFC and amyg, respectively) and a reference electrode located in vStr are shown for mesdopetam treatment, as compared to baseline and MK-801+vehicle. Two main effects are apparent. First, after MK-801 treatment, HFOs became more coherent (i.e. the lobes denoting the phase distributions became narrower in the circular plots) both within vStr and in the other structures, with close to zero phase differences in vStr but quite distinct phase difference for electrodes in the other structures (e.g. mPFC electrodes leading vStr with about π/8). Second, all three anti-psychotic treatments tended to reduce the MK-801-induced phase-locking. Pooling all recordings together and comparing changes in the density of the phase distributions induced by different treatments (changes in kappa-value, as defined by a circular normal distribution; σ^2^ ​= ​1/κ), it was clear that the decrease in phase-locking occurred on a global scale and for all three compounds (Fig. 5C). Because abnormal synchrony of HFOs has been observed following treatment with different psychotomimetic drugs, this reduction in abnormal coupling of HFOs induced by the anti-psychotic drugs may represent a reversal toward a more normal conscious state [52].

Since MK-801 induces an altered state of consciousness and at higher doses acts as a dissociative anesthetic, complexity measures that have been used to differentiate between normal and altered conscious states in the context of classical psychedelics drugs [55], as well as conscious and unconscious states in the context of general anesthesia [56], could potentially be of relevance to describe the brain state induced in this model of PD-P. Therefore, we next investigated to what extent the degree of order/complexity in the temporal structure of the recorded brain activity differed between the different treatments. To this aim, we analyzed neurophysiological brain states applying a metric based on permutation entropy, which has previously been shown to be particularly effective in differentiating between conscious and unconscious anesthetic states [57]. We focused on temporal entropy on a timescale shorter than the period of the HFOs, in order to avoid confounding effects of the oscillations. Interestingly, following MK-801 treatment, the temporal complexity of the signal was found to be significantly decreased in all recorded structures in the lesioned hemisphere, and often the antipsychotic drugs tended to reverse the neurophysiological state toward a more complex temporal structure, more resembling the baseline values (see e.g. OFC and vStr in Fig. 5E). However, in general, the changes in entropy measures were quite specific for each drug and brain structure suggesting these independent measures - which are based on LFP dynamics on a finer time scale than HFO oscillatory activity - may provide complementary information on each compound's specific pharmacological properties.

### Comparisons of drug-induced brain-states

**Fig. 6:**
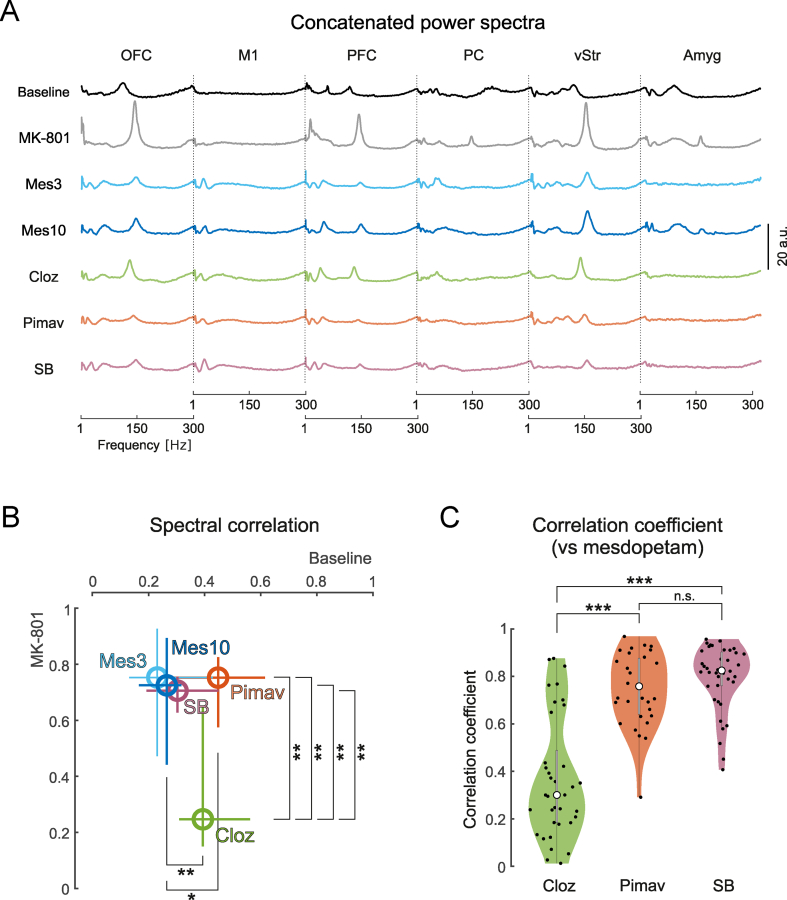
**Comparison of global brain states induced by mesdopetam, pimavanserin, clozapine, and the selective dopamine D3 antagonist SB-227011-A**. A) Concatenated LFP power spectra of six structures (1–300Hz), for vehicle and different treatments (mesdopetam, clozapine, pimavanserin, and SB-227011-A in combination with MK-801). Spectra are normalized to the arrhythmic background. B) Correlation analyses of spectra against baseline and vehicle for different treatments. The circles and whiskers show median values with 25th and 75th percentiles of the corresponding correlation coefficients against vehicle (vertical axis) and baseline (horizontal axis). Statistical comparisons are given for significant differences only (∗p ​< ​0.05; ∗∗p ​< ​0.01, Wilcoxon rank sum test). C) Pairwise spectral correlation coefficients between mesdopetam and the other treatments (mesdopetam 3 ​mg/kg and 10 ​mg/kg were merged for this analysis; Kruskal-Wallis test, ∗∗∗p ​< ​0.001; n.s. p ​> ​0.05).

Comparisons of the features of the recorded LFPs discussed above suggest that mesdopetam shares certain treatment effects with both clozapine and pimavanserin (e.g. reduction of HFOs in PFC and a global functional decoupling in the HFO frequency range). However, in other respects it was evident that the three tested compounds had different response profiles (e.g. changes in HFO peak frequency and complexity of the LFP signals recorded in different brain structures). A reasonable explanation for these differences is that different neuromodulatory systems are differentially affected by the three drugs. Thus, to test the hypothesis that mesdopetam acts primarily via antagonism of the dopamine type 3 receptors (D3R), we next performed a direct comparison of the global brain state induced, not only comparing mesdopetam to pimavanserin and clozapine but also to the selective D3R antagonist SB-277011-A [20]. To attain a description of the global physiological state that emphasizes oscillatory phenomena, we first normalized the LFP power density spectra to the non-oscillatory component for each treated state and brain structure. We then considered the global treatment effect of each antipsychotic compound to be represented by the spectral content across multiple recorded brain structures in a wide frequency band (1–300 ​Hz; Fig. 6A). The global state induced by the two doses of mesdopetam was then quantitatively compared to the three reference drugs by correlation of the spectral density of each of the distributions to the baseline state and MK-801 treated state, respectively (Fig. 6B). This comparison of global similarity demonstrated that the neurophysiological brain state induced by mesdopetam (both for 3 and 10 ​mg/kg) was most similar to that induced by SB-277011-A, whereas the largest differences were found in comparison to clozapine. Specifically, when directly assessing the pairwise global similarity score, by comparing spectral correlations of mesdopetam (3 and 10 ​mg/kg pooled together for better statistical power) and each of the other compounds in a pairwise manner, the greatest similarities were found for SB-277011-A and pimavanserin, whereas clozapine clearly displayed different spectral characteristics (Fig. 6C).

## Discussion

By comparing brain-wide physiological brain states induced by clozapine, pimavanserin and the novel prospective anti-psychotic compound mesdopetam in a rodent model of PD-P, we have here characterized the treatment profiles of these substances on a level of detail that has previously not been accessible. In the present study, behavioral readouts showed relatively large variability, and only marginally helped elucidating the treatment effects of the tested compounds. More importantly, the use of specialized techniques based on large-scale multi-structure recordings giving access to systems-level characterizations of brain states resulted in a comprehensive description pointing to a number of both shared and drug-specific physiological treatment effects. In agreement with previous reports investigating the neurophysiological responses to psychotomimetic drugs, we found that the state of experimental psychosis was associated with distinct HFOs in a number of cognitive-limbic structures [[46], [47], [48], [49], [50], [51], [52]]. Interestingly, all three types of antipsychotics tested were found to reduce these oscillations in mPFC. Perhaps equally important, the synchrony of these HFOs across different brain structures was also significantly reduced, indicating that alterations of systems-level network dynamics may be an important therapeutic mechanism.

However, for other features of the recorded neurophysiological signals, the compounds differed substantially, for example with respect to HFO peak frequency and signal complexity observed in different brain structures. Overall, when comparing the drug-treated brain states - on a global scale - it was evident the compounds showed graded difference to each other, indicating mesdopetam, at both of the tested doses, is functionally more closely related to the selective D3R antagonist SB-277011-A, and to pimavanserin, than to clozapine. It can be speculated, that in the rat model of PD (with l-DOPA priming) studied here, maladaptive cellular adaptations have taken place, involving for example D3R sensitization and D1R-D3R cooperativity [58], that may underlie the observed propensity for excessive HFOs and abnormal functional coupling. In this situation (most closely resembling clinical later-stage PD with dyskinesia), pharmacological suppression of D3R-signaling could help ameliorate side effects of chronic levodopa treatment, including symptoms of PD-P. Regarding psychosis in general, modulation of the D2/D3 receptor family, either antagonism or partial agonism, represents an established pharmacological treatment principle. PD-P in particular, has been described as a state of imbalance between serotonin and dopamine systems, possibly linked to specific alterations in cortical 5-HT2A-receptors, with downstream effects leading to excessive limbic dopamine signaling, which could be a substrate for antipsychotic effects of mesdopetam in PD-P [59]. In, in future studies, the direct effects of l-DOPA on brain states of experimental psychosis deserve to be investigated in greater detail, including in the context of antipsychotic treatment, given the tendency for dopamine replacement therapy to aggravate symptoms of PD-P [3,5].

While these neurophysiological characterizations in an animal model of PD-P clearly offer several deeper insights into the treatment effects of these drugs, it should be acknowledged that the altered state of consciousness induced by NMDA antagonists such as MK-801 does not capture all characteristics of a psychotic episode. Perhaps most importantly, the subject is usually aware that the altered state of consciousness is drug-induced in the former case whereas psychotic patients cannot link their experiences to their medical condition. Keeping this limitation in mind, psychotomimetic drugs nevertheless remain a viable approach to studying putative antipsychotic therapies given the many shared components between the two states, such visual hallucinations, and delusions [60]. Moreover, EEG studies in human psychosis have often reported considerable variability between subjects. For example, in studies of signal complexity, divergent patterns of change have been observed depending on factors such as stage and type of schizophrenia, resting vs. active conditions, etc. [61].

In conclusion, our results indicate that mesdopetam shares certain key treatment effects with existing antipsychotic treatments for PD-P and consequently may have the potential to become a novel antipsychotic treatment option in this condition. At the same time, the specific response patterns of the different drugs could help elucidate their respective mechanisms of action and help predict potential side effects. In a broader perspective, studies linking the effects of pharmacological treatments on neurophysiological brain states [19] to neurochemical properties as well as behavioral data [17], has the potential not only to speed up drug development but also to fill a significant knowledge gap in neuroscience by helping to merge the disparate descriptions of brain functions that have emerged from the different scientific traditions of neurochemical and neurophysiological studies.

## Author contributions

Tiberiu Loredan Stan and Abdolaziz Ronaghi performed the experiments.

Sebastian A. Barrientos, Pär Halje, Luciano Censoni, Azat Nasretdinov developed software and analyzed the data.

Sebastian A. Barrientos, ​Luciano Censoni, Emilio Garro-Martínez and Evgenya Malinina took part in methodological development and behavioral analyses.

Stephan Hjorth, Peder Svensson, Susanna Waters were part of funding acquisition and experimental design.

Kristoffer Sahlholm took part in experimental design.

Per Petersson was part of funding acquisition and experimental design and wrote the draft of the manuscript.

All authors contributed to the editing of the final version of the manuscript.

## Declaration of competing interest

The authors declare the following financial interests/personal relationships which may be considered as potential competing interests: Stephan Hjorth, Peder Svensson, Susanna Waters are employed by IRL AB and hold stock in IRL AB. Remaining authors declare that they have no known competing financial interests or personal relationships that could have appeared to influence the work reported in this paper.
