# Supplementary material for: Neurophysiological treatment effects of mesdopetam, pimavanserin and clozapine in a rodent model of Parkinson's disease psychosis
Source: Neurotherapeutics. 2024 Feb 16;21(2):e00334. doi: 10.1016/j.neurot.2024.e00334 (PMC10937958; doi:10.1016/j.neurot.2024.e00334)
Supplement: Multimedia component 1 [file mmc1.pdf]

a

	A	B	C	D	E	F	G	H
agranular insular cortex, dorsal part	0	0	0	0	0	0	0	1
agranular insular cortex, posterior part	0	0	0	0	0	0	1	0
agranular insular cortex, ventral part	4	0	0	5	4	0	0	6
caudate putamen (striatum)	6	8	18	24	12	9	8	10
cingulate cortex, area 1	1	0	0	4	1	1	1	2
cingulate cortex, area 2	0	0	2	1	0	1	0	0
claustrum	0	0	0	0	0	1	0	0
ventral part of claustrum	0	0	0	1	0	0	0	0
globus pallidus	0	0	0	1	0	0	0	0
nucleus of the brachium of the inferior colliculus	0	3	0	0	0	0	0	0
external cortex of the inferior colliculus	0	0	1	0	0	0	0	0
brachium of the inferior colliculus	0	2	0	0	0	0	0	0
primary motor cortex	13	6	0	8	2	10	8	1
secondary motor cortex	1	6	0	2	0	0	4	1
mediodorsal thalamic nucleus, central part	0	0	0	1	0	0	7	0
mediodorsal thalamic nucleus, lateral part	0	0	0	2	0	1	6	0
mediodorsal thalamic nucleus, medial part	0	0	0	0	0	1	2	0
medial geniculate nucleus, dorsal part	1	0	0	0	0	0	0	0
medial geniculate nucleus, medial part	0	0	0	0	0	0	1	0
medial geniculate nucleus, ventral part	0	0	0	1	0	3	2	0
medial preoptic area	0	0	1	0	0	0	0	0
lateral orbital cortex	14	14	0	6	2	1	16	0
ventral orbital cortex	0	2	0	1	0	3	2	0
infralimbic cortex	0	0	6	0	0	1	2	0
prelimbic cortex	10	9	0	1	0	6	6	3
anterior pretectal nucleus	1	0	0	0	0	0	0	0
anterior pretectal nucleus, dorsal part	2	0	0	0	0	0	0	0
lateral parietal association cortex	8	6	2	3	0	0	2	3
parietal cortex, posterior area, dorsal part	3	9	4	4	0	0	1	0
parietal cortex, posterior area, rostral part	0	0	3	0	1	0	0	0
retrosplenial granular cortex, c region	0	0	0	0	0	0	0	1
primary somatosensory cortex, barrel field	0	0	1	5	3	0	0	0
primary somatosensory cortex, dysgranular zone	0	0	0	0	0	1	0	0
primary somatosensory cortex, forelimb region	0	2	1	7	7	5	2	7
primary somatosensory cortex, trunk region	4	0	0	3	0	0	0	2
primary somatosensory cortex, upper lip region	0	0	1	0	0	2	0	0
secondary somatosensory cortex	0	0	0	1	0	0	1	0
intermediate gray layer of the superior colliculus	0	1	0	0	0	0	0	0
intermediate white layer of the superior colliculus	2	2	0	0	0	0	0	0
brachium of the superior colliculus	2	0	0	0	0	0	0	0
substantia nigra, lateral part	0	0	0	0	0	1	0	0
substantia nigra, reticular part	0	0	0	0	0	0	0	1
accumbens nucleus, core	6	7	0	0	1	6	8	5
accumbens nucleus, shell	2	5	0	0	4	3	0	3
islands of Calleja	0	0	4	0	0	0	0	0
lateral accumbens shell	0	0	0	0	0	0	0	5
secondary visual cortex, lateral area	0	1	0	0	0	0	0	0
ventral pallidum	0	0	3	0	11	0	0	0
amygdalohippocampal area, anterolateral part	0	0	0	0	0	0	0	1
amygdalohippocampal area, posterolateral	0	0	0	0	0	0	0	1
amygdalohippocampal area, posteromedial part	2	0	0	0	0	0	0	2
amygdalopiriform transition area	6	5	0	2	0	0	0	1
basolateral amygdaloid nucleus, anterior part	0	0	0	1	0	2	1	0
basolateral amygdaloid nucleus, posterior part	2	3	0	1	0	6	0	3
basolateral amygdaloid nucleus, ventral part	0	0	2	0	0	0	0	1
basomedial amygdaloid nucleus, posterior part	2	3	0	0	0	5	0	2
interstitial nucleus of the posterior limb of the anterior commissure	0	0	0	0	0	0	0	2
lateral amygdaloid nucleus, ventromedial part	4	0	0	0	0	0	3	0
bed nucleus of the stria terminalis, intraamygdaloid division	0	0	0	0	0	0	0	2
lateral habenular nucleus, medial part	0	0	0	0	1	0	0	0
medial habenular nucleus	0	0	0	0	0	1	0	0
field CA2 of the hippocampus	0	0	0	0	2	0	0	0
granular layer of the dentate gyrus	0	0	0	1	0	0	0	0
lacunosum moleculare layer of the hippocampus	0	0	0	0	0	0	0	1
molecular layer of the dentate gyrus	0	0	0	0	2	0	0	1
stratum lucidum of the hippocampus	0	0	0	0	0	0	1	0
subiculum, transition area	0	0	0	1	0	0	0	0
field CA1 of the hippocampus	3	0	0	2	1	0	0	0
field CA3 of the hippocampus	0	0	1	1	0	0	1	1
ectorhinal cortex	0	0	0	1	0	0	0	0
oriens layer of the hippocampus	0	0	6	0	5	1	3	1
perirhinal cortex	0	0	0	4	0	0	0	0
polymorph layer of the dentate gyrus	0	0	1	0	4	0	0	0
radiatum layer of the hippocampus	0	1	0	0	0	1	3	1
ventral intermediate entorhinal cortex	0	1	0	0	0	0	0	0
ventral subiculum	3	0	0	0	0	0	2	4
lateral olfactory tract	0	0	11	0	0	0	0	0
medial forebrain bundle	0	0	1	0	0	0	0	0
layer 2 of cortex	0	0	1	0	2	0	0	0
layer 3 of cortex	0	0	6	0	3	0	0	0
dorsal endopiriform nucleus	3	0	0	1	0	0	2	0
intermediate endopiriform nucleus	0	0	0	0	3	0	0	0
posterolateral cortical amygdaloid nucleus	0	0	0	0	3	0	0	0
posterolateral cortical amygdaloid nucleus, layer 1	0	0	0	0	0	1	0	0
posteromedial cortical amygdaloid nucleus	0	0	8	0	5	0	0	0
piriform cortex	0	0	0	2	0	0	3	0
piriform cortex, layer 1	0	0	0	0	11	4	0	5
piriform cortex, layer 1a	0	2	0	0	0	0	0	0
ventral endopiriform nucleus	0	0	0	0	0	3	0	0
dorsal peduncular cortex	0	0	0	0	0	1	0	0
lateral septal nucleus, intermediate part	0	0	0	0	1	0	0	2
medial septal nucleus	0	0	0	0	0	0	0	1
nucleus of the vertical limb of the diagonal band	0	0	0	0	0	0	0	1
fimbria of the hippocampus	0	0	0	0	1	3	1	0
anteromedial thalamic nucleus	0	0	0	0	0	0	0	1
anteroventral thalamic nucleus, dorsomedial part	0	0	0	5	0	0	0	0
anteroventral thalamic nucleus, ventrolateral part	0	0	0	1	0	1	0	0
angular thalamic nucleus	0	0	0	1	0	0	1	0
centrolateral thalamic nucleus	0	0	0	1	0	0	0	0
central medial thalamic nucleus	0	0	0	0	8	4	0	3
interanteromedial thalamic nucleus	0	0	0	0	0	1	0	0
laterodorsal thalamic nucleus, ventrolateral part	0	0	0	3	0	0	0	0
lateral posterior thalamic nucleus, mediocaudal part	0	0	0	0	0	1	0	0
paracentral thalamic nucleus	0	0	0	1	0	2	0	0
posterior intralaminar thalamic nucleus	0	0	2	0	1	0	0	1
posterior thalamic nuclear group	0	0	0	0	0	0	0	3
posterior thalamic nuclear group, triangular part	0	0	0	0	0	0	0	2
reticular thalamic nucleus	0	0	3	0	0	1	0	0
subgeniculate nucleus	0	0	0	0	0	1	0	0
ventral anterior thalamic nucleus	0	0	0	0	0	0	0	2
ventral geniculate nucleus, layer 1	0	0	0	1	0	0	0	2
ventrolateral thalamic nucleus	0	0	0	1	2	0	0	3
ventromedial thalamic nucleus	0	0	0	0	1	0	0	0
stria medullaris of the thalamus	0	0	0	0	1	0	0	0

Animal

b

	A	B	C	D	E	F	G	H
agranular insular cortex	4	0	0	5	4	0	1	7
amygdala	16	11	2	4	0	13	4	15
claustrum	0	0	0	1	0	1	0	0
dorsal hippocampus	0	0	8	3	8	0	4	2
dorsal striatum	6	8	18	24	12	9	8	10
globus pallidus	0	0	0	1	0	0	0	0
habenula	0	0	0	0	1	1	0	0
inferior colliculus	0	5	1	0	0	0	0	0
medial forebrain bundle	0	0	1	0	0	0	0	0
medial geniculate nucleus	1	0	0	1	0	3	3	0
mediodorsal thalamic nucleus	0	0	0	3	0	2	15	0
olfactory cortex	3	2	14	3	27	8	5	5
olfactory tract	0	0	11	0	0	0	0	0
orbitofrontal cortex	14	16	0	7	2	4	18	0
parietal association cortex	11	15	9	7	1	0	3	4
prefrontal cortex	10	9	6	1	0	7	8	3
preoptic area	0	0	1	0	0	0	0	0
pretectum	3	0	0	0	0	0	0	0
primary motor cortex	13	6	0	8	2	10	8	1
primary somatosensory cortex	4	2	3	15	10	8	2	9
secondary motor cortex	1	6	0	2	0	0	4	1
secondary somatosensory cortex	0	0	0	1	0	0	1	0
secondary visual cortex	0	1	0	0	0	0	0	0
septum	0	0	0	0	2	4	1	4
substantia nigra, reticular part	0	0	0	0	0	1	0	1
superior colliculus	4	3	0	0	0	0	0	0
thalamus	0	0	5	14	13	11	1	17
ventral hippocampus	6	2	0	7	6	2	6	7
ventral pallidum	0	0	3	0	11	0	0	0
ventral striatum	8	12	5	0	5	9	8	13

Animal
