# Supplementary figures and images for: Neurophysiological treatment effects of mesdopetam, pimavanserin and clozapine in a rodent model of Parkinson's disease psychosis
Source: Neurotherapeutics. 2024 Feb 16;21(2):e00334. doi: 10.1016/j.neurot.2024.e00334 (PMC10937958; doi:10.1016/j.neurot.2024.e00334)

a

Detections, Gamma

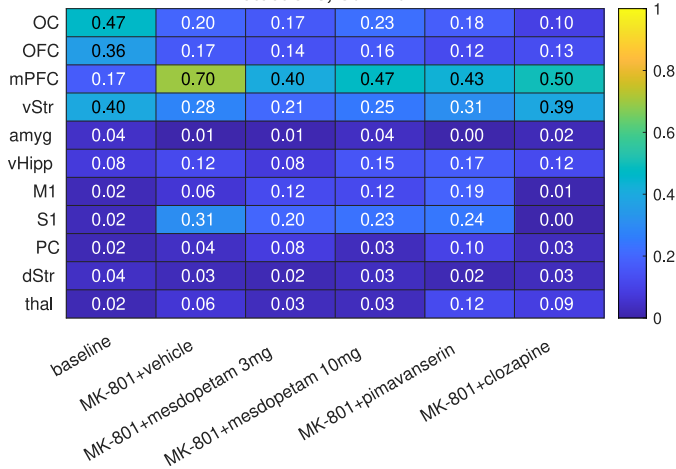

b

Bandpower, Gamma

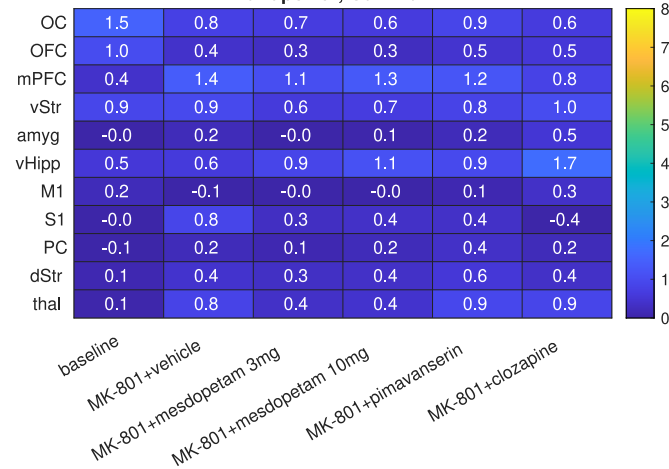

c

Peak frequency, Gamma

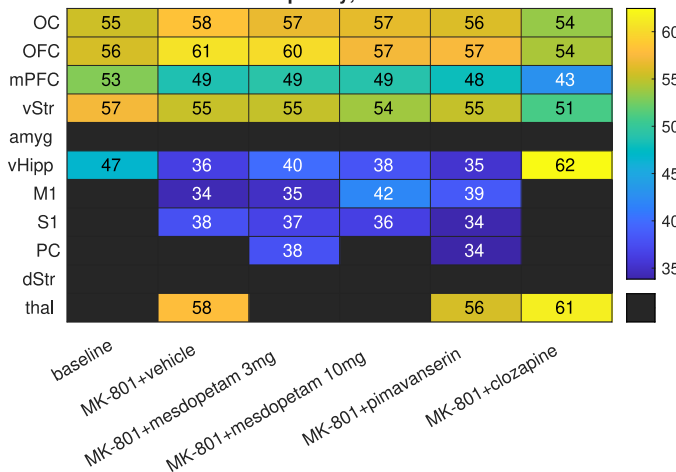

Supplement: Multimedia component 2 [file mmc2.pdf]

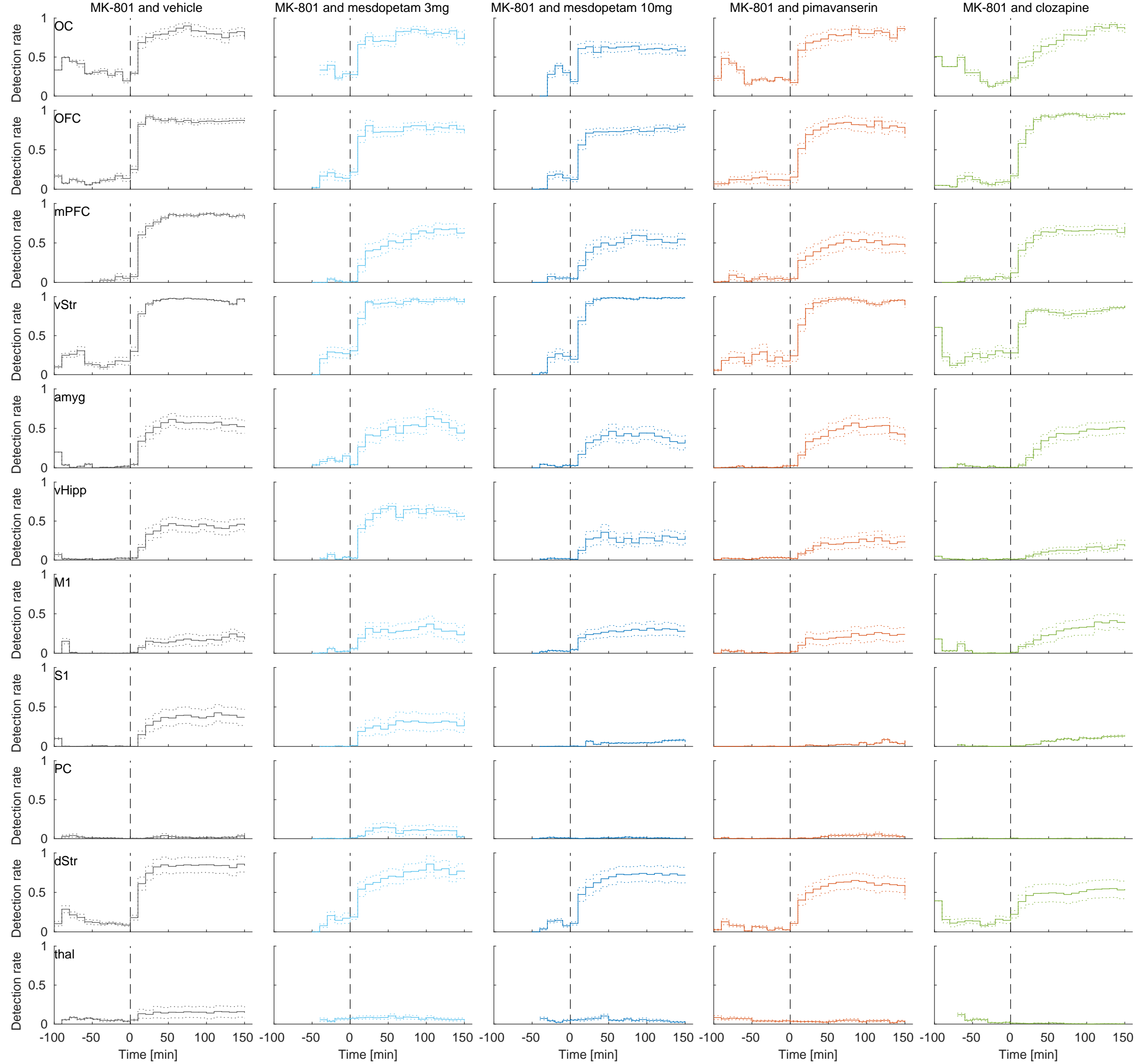

Supplement: Multimedia component 3 [file mmc3.pdf]

a

Detections, 150Hz

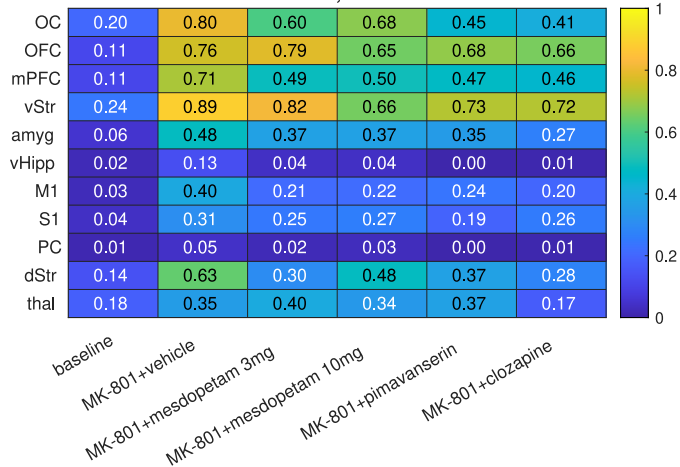

b

Bandpower, 150Hz

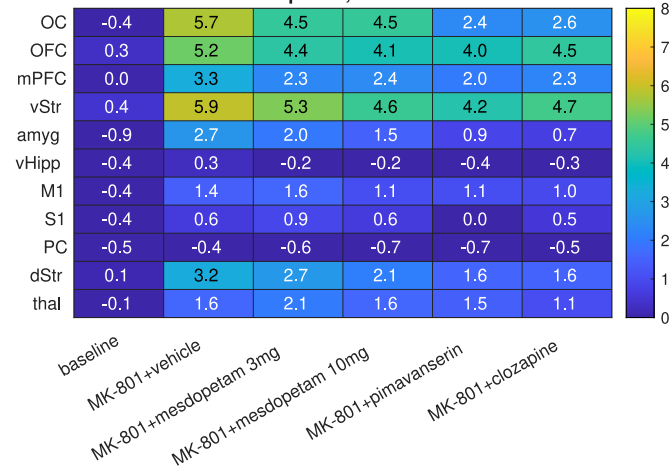

c

Peak frequency, 150Hz

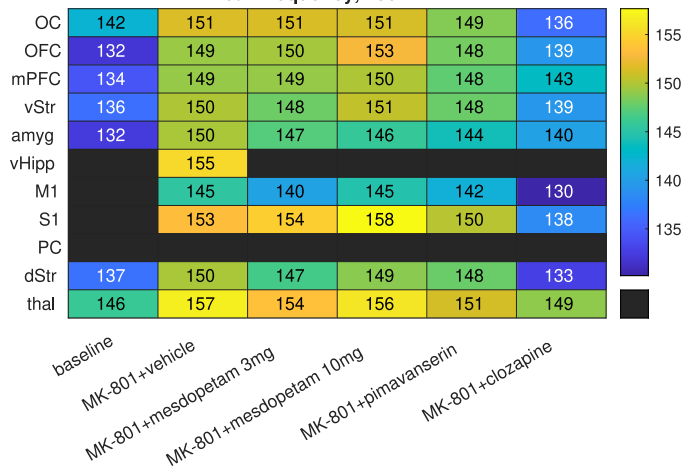

Supplement: Multimedia component 4 [file mmc4.pdf]
