# Supplementary material for: Neurophysiological treatment effects of mesdopetam, pimavanserin and clozapine in a rodent model of Parkinson's disease psychosis
Source: Neurotherapeutics. 2024 Feb 16;21(2):e00334. doi: 10.1016/j.neurot.2024.e00334 (PMC10937958; doi:10.1016/j.neurot.2024.e00334)
Supplement: Multimedia component 5 [file mmc5.docx]

**Description of Supplementary Data and Figures**

**Supplementary Data – Summary of implantation coordinates for each of the 634 validated electrode tips included in the study.** Columns refer to Wire-ID, Animal-ID, Hemisphere (right = lesioned), Location - using the functional grouping applied in the manuscript, Location - according to Paxinos and Watson Atlas of the rat brain, Location confirmed by CT (true=’1’), mediolateral coordinate, anteroposterior coordinate, dorsoventral coordinate (all with reference to Bregma).

**Supplementary Figure 1 – Summary of electrode locations in each rat. A)** Table showing the number of electrode tips determined to be located in each structure, as delineated in the Paxinos and Watson Atlas of the rat brain (columns denote animal identity, [A-H]). **B)** Table showing the number of electrode tips in each structure following functional grouping. Colors denote relative tip density (min-max) across all structures.

**Supplementary Figure 2 – Pharmacological treatment effects on gamma-band oscillations (30-70 Hz) across all recordings and animals.** **A)** Detection rates. **B)** Band power (dB) **C)** Peak frequency.

**Supplementary Figure 3 – Average time evolution of HFO detection rates across all recordings divided by structure (rows) and condition (columns) for the lesioned hemisphere.** Note similarities in the temporal patterns of HFO detection rates, but with relative reductions following antipsychotic treatment compared to MK-801+vehicle in certain structures (e.g. mPFC). Vertical dashed lines mark MK-801 injection. Dotted lines denote the standard error of the mean for each time bin.

**Supplementary Figure 4 – Pharmacological treatment effects on HFOs in the intact hemisphere. A)** Detection rates. **B)** Band power (dB). **C)** Peak frequency. Areas with no data are marked in black.
